# Pharyngeal pumping phenotypes of common fluorescently-tagged *C. elegans* strains

**DOI:** 10.17912/micropub.biology.000267

**Published:** 2020-06-09

**Authors:** Rosemary Bauer, Andy Golden

**Affiliations:** 1 Laboratory of Biochemistry and Genetics, National Institute of Diabetes and Digestive and Kidney Diseases, NIH, Bethesda, MD 20892

**Figure 1:**
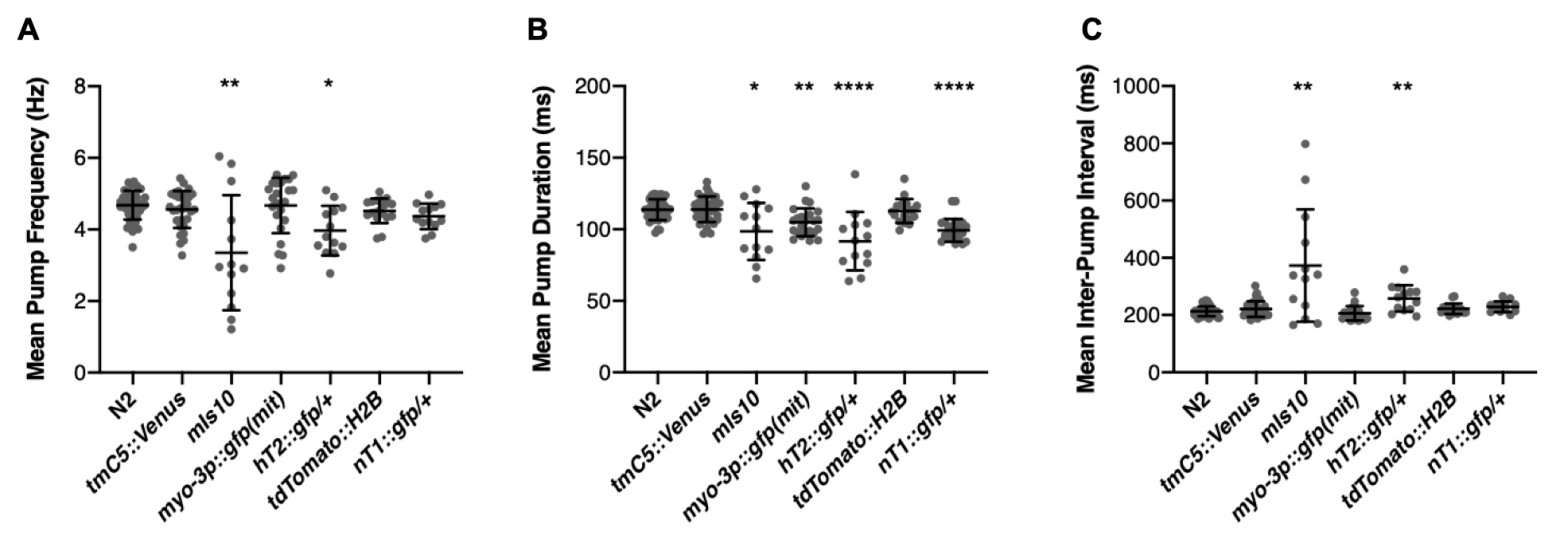
*C. elegans* strains commonly used as chromosomal balancers have moderate to severe alterations in (A) pharyngeal pump frequency, (B) pump duration, and (C) inter-pump interval (IPI) compared to N2. In particular, the chromosome V balancer *mIs10* exhibits a dramatic reduction in pump frequency (Kruskal-Wallis test, Dunn’s multiple comparisons, p= 0.006). The reciprocal translocation *hT2::gfp*(I;III) also causes changes in pump frequency, duration, and IPI (Kruskal-Wallis test, Dunn’s multiple comparisons, p= 0.013, p<.0001, p= 0.007). Additionally, the integrated mitochondria-localized muscular GFP *myo-3*p::gfp(mit) and the reciprocal translocation *nT1::gfp*(IV;V) cause statistically significant but physiologically moderate changes in pump duration (Kruskal-Wallis test, Dunn’s multiple comparisons, p= 0.006, p< 0.0001). Bars represent mean ± 1 SD. Average n=23.

## Description

Genetic balancer strains are frequently used to maintain populations of *C. elegans* mutants that would otherwise die as homozygotes. Additionally, fluorescently-marked strains can be used to identify cross-progeny for use in assays where heterozygotes are of particular interest. In our case, we wanted to utilize a fluorescently-marked but otherwise wildtype strain to obtain marked cross-progeny from a mating. Our mutant of interest displayed altered pharyngeal pumping, so we wanted to assess heterozygotes in the same assay and establish the recessive nature of the phenotype. We happened to have the TY4236 strain growing in the lab, which not only expresses bright pharyngeal and gut GFP from the *mIs10* insertion but also contains *him-8(e1489)*, making the experiment quite simple. Due to the bright GFP signal in the pharynx we decided to assess the pharyngeal pumping of the strain itself before using it as a marker in an experiment that heavily implicates the pharynx.

To our surprise, this otherwise-healthy strain displayed highly variable pharyngeal pump frequency, decreased average pump duration, and increased average inter-pump interval (IPI) when compared to N2 (Fig 1A-C). This prompted us to test multiple other options, including some that also express GFP in the pharynx (*tmC5::Venus, hT2::gfp*, *nT1::gfp*) as well as two that don’t [*myo-3p::gfp::GFP(mit)*, *tdTomato::H2B*]. Two balancers with bright pharyngeal GFP, *hT2::gfp* and *nT1::gfp*, displayed moderate pumping phenotypes even as heterozygotes (Fig 1A-C). Interestingly, the pharyngeal pumping phenotypes do not correlate exactly with fluorescence localization; *tmC5::Venus* was completely unaffected and *myo-3p::gfp(mit) myo-3*::GFP(mito) had slightly but significantly reduced mean pump duration (Fig 1B). Besides N2, each strain was only assayed once and therefore these results should be treated as preliminary. Additionally, since these strains were all created in different labs across the world and in some cases across the span of many years, it is possible that the pumping phenotype is due, in part, to the genetic background of the strain and not entirely due to the insertion of a fluorescent tag.

Pharyngeal pumping phenotypes can indicate upstream nervous system or muscular alterations and can lead to downstream outcomes like altered metabolism and lifespan (Raizen *et al.*, 1995). Regardless of the experimenter’s interest in characterizing pharyngeal pumping, these fluorescent strains should be assessed for possible subtle phenotypes that could potentially confound the main phenotype of interest.

## Methods

Electropharyngeograms

Fifteen gravid hermaphrodites of each genotype were placed on OP50-seeded MYOB plates and allowed to lay embryos for 6 hours before removal to establish a synchronized F1 population. After 3 days the synchronized population was washed off with M9, washed 3X, then incubated in 10mM 5HT for at least 20 minutes. Pharyngeal pumping was then recorded using the NemaMetrix ScreenChip system (Eugene, OR). All genotypes were assayed as homozygotes with the exception of *nT1::gfp* and *hT2::gfp*, which are homozygous lethal. Strains were grown at 20°C at all times except immediately leading up to and during the experiment. Strains were assayed across 5 different days; N2 was included on each of the different days to ensure any variability was not due to environmental conditions. Outliers were identified by ROUT with Q=0.5%. Since one or more strains in each data set did not pass the Anderson-Darling test for normality, strains were compared by Kruskal-Wallis with Dunn’s multiple comparison test.

## Reagents

Strains

**Table:** 

Strain Name	Genotype	Chromosomal Location	Fluorescence Localization	Reference
FX30140	*tmC5 [In(C01B10.3 eak-7 In(mec-3 unc-31) tmIs1220 (myo-2p::Venus)]*	IV	pharynx	Dejima, *et al.*, 2018
TY4236	*him-8(e1489)*; *mIs10* [*myo-2p::gfp* + *pes-10p::gfp + gut-promoter::gfp*]	V	pharynx, gut	Mark Edgley & Don Riddle, personal communication
SJ4103	*zcIs14 [myo-3p::gfp(mit)]*	??	body wall muscle	Benedetti *et al.*, 2006
AG589	*unc-38(x20) dpy-5(e61) / hT2******[bli-4(e937) let-?(q782) qIs48 (myo-2p::gfp; pes-10p::gfp; ges-1p::gfp)]; him-8(e1489)*	I, III	pharynx	McKim, *et al.*, 1993; Judith Kimble, personal communication
EG7919	*unc-119(ed3) III; oxTi584[eft-3p::tdTomato::H2B(his-58)::unc-54 3’UTR + Cbr-unc-119(+)]*	IV	pan-nuclear	Frøkjær-Jensen, *et al.*, 2014
JK2958	*dpy-11(e224) unc-42(e270) V / nT1 [qIs51 (myo-2p::gfp; pes-10p::gfp; F22B7.9p::gfp)]*	IV, V	pharynx, gut	Ferguson & Horvitz, 1985; Judith Kimble, personal communication
